# The dual role of APOE ε4 allele in cerebral small vessel disease: independent genetic effects and effect modification of traditional risk factors

**DOI:** 10.3389/fneur.2026.1778289

**Published:** 2026-08-05

**Authors:** Feng Liu, Yulan Gou

**Affiliations:** 1 Wuhan Hospital of Traditional Chinese and Western Medicine, Wuhan, China; 2 Department of Neurology, Wuhan No.1 Hospital, Wuhan, Hubei, China

**Keywords:** apolipoprotein E gene, cerebral small vessel diseases, effect modifier, gene–environment interaction, phenotype

## Abstract

**Background:**

The association between apolipoprotein E (APOE) ε4 and cerebral small vessel disease (CSVD) remains incompletely understood, with inconsistent evidence across imaging phenotypes and limited investigation into gene–environment interactions.

**Method:**

This cross-sectional study included 728 patients with chronic lacunar infarcts who underwent 3 T brain MRI. CSVD burden was quantified using a study-specific composite score (range 0–6), integrating white matter hyperintensities (WMH), cerebral microbleeds (CMBs), and enlarged perivascular spaces (EPVS). APOE ε4 carriers (n = 139) were compared with non-carriers (n = 589). Ordinal logistic regression and Firth‘s penalized logistic regression (for EPVS) were used to assess the independent effect of APOE ε4 on CSVD markers. Multiplicative interaction terms (APOE ε4 × smoking, APOE ε4 × alcohol use) were added to the fully adjusted models, with *p* values adjusted using the Holm-Bonferroni method. Sensitivity analyses excluded APOE ε2 carriers.

**Results:**

APOE ε4 was independently associated only with periventricular WMH burden (*β* = 0.411, 95% CI: 0.046–0.776, *p* = 0.027), with no significant associations for global CSVD burden, total WMH, deep WMH, CMBs, or EPVS. Significant interactions were observed for APOE ε4 × smoking on global CSVD burden, CMBs, total WMH, and PWMH (adjusted *p* < 0.05), and for APOE ε4 × alcohol use on global CSVD burden and CMBs (adjusted *p* < 0.05). These findings remained robust after excluding APOE ε2 carriers.

**Conclusion:**

APOE ε4 exhibits both an independent genetic effect—selectively associated with periventricular white matter injury—and a modifying effect on the associations of smoking and alcohol use with specific CSVD phenotypes. These findings highlight the phenotypic specificity of APOE ε4’s dual role in CSVD and support the integration of genetic background with modifiable risk factors for precision risk stratification.

## Introduction

Cerebral small vessel disease (CSVD) accounts for approximately 25% of ischemic strokes and represents the leading cause of primary intracerebral hemorrhage and vascular cognitive impairment in the elderly. It can be non-invasively assessed using conventional MRI. According to the latest neuroimaging criteria, CSVD encompasses white matter hyperintensities (WMH), enlarged perivascular spaces (EPVS), cerebral microbleeds (CMBs), lacunar infarcts, and brain atrophy (1–3).

Although the mechanisms linking APOE genotype to CSVD remain incompletely understood, accumulating evidence suggests that cerebral microvascular burden may accelerate cognitive decline by promoting amyloid deposition or increasing susceptibility to amyloid-related pathologies (3–5). APOE is well known to regulate amyloid-*β* metabolism and accumulation. More recently, the role of APOE in CSVD pathogenesis has expanded beyond amyloid-*β* to include tau neurofibrillary tangles, glial activation, and blood–brain barrier disruption (6–8).

However, it remains unclear whether APOE is associated with the full spectrum of CSVD imaging markers. Most earlier studies on APOE and CSVD were conducted in patients with dementia, depression, or other neurological conditions. A meta-analysis by Schilling et al. addressed this limitation by including population-based cohorts (9). Nevertheless, a critical gap persists: that meta-analysis focused exclusively on WMH and CMBs, excluding other core CSVD markers such as lacunar infarcts and EPVS, which are essential for capturing the heterogeneity of CSVD.

To address this gap, the present study systematically investigated the association between APOE ε4 and the complete spectrum of CSVD imaging phenotypes in a cohort of patients with recent lacunar ischemic stroke. Specifically, we sought to answer three core research questions:

(1) Is the APOE ε4 allele independently associated with global CSVD burden and its individual imaging phenotypes (WMH, CMBs, and EPVS)?
(2) Does the association between APOE ε4 and CSVD exhibit specificity across different imaging phenotypes?
(3) Does APOE ε4 interact with traditional vascular risk factors (e.g., hypertension, smoking) in their association with CSVD imaging metrics?

(4) By addressing these questions, this study describes cross-sectional associations between APOE ε4 and CSVD imaging phenotypes, and explores potential statistical interactions with traditional vascular risk factors. These findings provide descriptive evidence that may inform future hypothesis-driven research and precision risk stratification.

## Materials and methods

### Participants

A total of 818 patients were initially recruited from the Cerebral Small Vessel Disease Unit of the Department of Neurology at the Traditional Chinese and Western Medicine Hospital of Wuhan, Tongji Medical College, Huazhong University of Science and Technology, between July 2024 and September 2025. These patients had a history of lacunar ischemic stroke and underwent examination with a 3 T MRI scanner during this period. For this study, we excluded individuals with incomplete clinical data (*n* = 18), those without definite chronic lacunar infarcts after independent adjudication by three experienced clinicians (*n* = 60), and—due to potential confounding effects associated with the APOE genotype—carriers of the APOE ε2/ε4 genotype (*n* = 12). After these exclusions, a total of 728 patients were finally included in the analysis.

### Inclusion criteria

1. Patients aged 18 years or older;
2. Imaging confirmation: Presence of one or more chronic lacunar infarcts on 3 T MRI, defined according to STRIVE-2 (1)criteria as round or ovoid lesions, 3–15 mm in diameter, located in the territory of a perforating artery, appearing as central cerebrospinal fluid-like hypointensity on T2-FLAIR with or without a hyperintense rim, and no restricted diffusion on DWI (to exclude acute infarcts);
3. Clinical history: A documented history of clinical lacunar stroke, or imaging-detected lacunes that correspond to current residual neurological deficits/signs (e.g., hemiparesis, sensory disturbance, dysarthria);
4. Completeness of clinical data: Availability of comprehensive clinical data, including demographic information, vascular risk factors, and laboratory results Clinical Data Collection and Definition;
5. Completeness of imaging sequences: Availability of full MRI sequences (e.g., T1-weighted, T2-weighted, FLAIR, DWI, SWI) for comprehensive assessment of cerebral small vessel disease markers.

### Exclusion criteria

1 Evidence of Acute Infarction: Presence of acute or subacute infarcts confirmed by hyperintense signals on diffusion-weighted imaging (DWI) sequences.
2 Alternative Etiologies for Stroke: Evidence of stroke mechanisms other than cerebral small vessel disease, including:

(a) Cardioembolic sources (e.g., atrial fibrillation, valvular heart disease);
(b) Large-artery atherosclerosis with ≥50% luminal stenosis in the relevant extracranial or intracranial arteries;
(c) Cortical infarcts or watershed infarcts.

3 Other Neurological or Systemic Conditions: Presence of other neurological diseases or systemic disorders that could potentially affect brain structure or confound the assessment of CSVD markers, such as multiple sclerosis, traumatic brain injury, brain tumors, central nervous system infection, or a history of intracranial hemorrhage (excluding microbleeds).
4 Poor Imaging Quality: Insufficient MRI image quality (e.g., significant motion artifacts) that precluded reliable visual assessment of CSVD features.
5 Incomplete Data: Lack of essential clinical data required for the analysis of primary study variables.
6 Contraindications to MRI: Standard contraindications to 3 T MRI, such as the presence of incompatible metallic implants or severe claustrophobia.

#### Clinical data collection and definitions

Demographic and clinical characteristics of the patients were collected, including sex, age, and the presence of comorbidities such as hypertension, diabetes mellitus, hyperlipidemia, and atherosclerosis, as well as neuroimaging data. Traditional vascular risk factors (VRFs) were defined as follows: Hypertension was defined as a documented diagnosis of hypertension in clinical records or at hospital admission. Diabetes mellitus was defined as an HbA1c level ≥6.5% or a known diagnosis documented in clinical records. Hyperlipidemia was defined as a prior diagnosis recorded in clinical documentation. Smoking status and alcohol consumption were classified based on current use.

### APOE genotyping and stratification

APOE genotyping was performed using a commercially available Human APOE Genotyping Kit based on the fluorescent PCR-restriction fragment length polymorphism (PCR-RFLP) assay, with detailed methodology provided in Supplementary File 1. Individuals carrying at least one APOE ε4 allele (genotypes ε4/ε4 or ε4/ε3) were classified as APOE ε4 carriers, whereas those without any ε4 allele (i.e., ε2/ε2, ε2/ε3, or ε3/ε3) were classified as non-carriers. To eliminate potential confounding effects related to genetic heterogeneity, carriers of the APOE ε2/ε4 genotype were excluded from the analysis.

### 3 t MRI

The patients were scanned on a Siemens/MAGENTOM Vida 3.0 T MRI scanner in the Department of Radiology of Wuhan No.1 Hospital. The detailed imaging parameters were as follows:

1. T1-weighted sequence: Repetition time (TR) = 1800 ms, echo time (TE) = 20 ms, slice thickness = 5 mm, slice gap = 1 mm, field of view (FOV) = 240 × 240 mm, matrix = 256 × 256;
2. T2-weighted sequence: TR = 4,500 ms, TE = 100 ms, slice thickness = 5 mm, slice gap = 1 mm, FOV = 240 × 240 mm, matrix = 256 × 256;
3. FLAIR sequence: TR = 8,000 ms, TE = 120 ms, inversion time (TI) = 2000 ms, slice thickness = 5 mm, slice gap = 1 mm, FOV = 240 × 240 mm, matrix = 256 × 256;
4. SWI sequence: TR = 28 ms, TE = 20 ms, slice thickness = 2 mm, slice gap = 0 mm, FOV = 240 × 240 mm, matrix = 384 × 384.

### Neuroimaging assessment of CSVD

All MRI scans were independently evaluated by two neuroradiologists who were blinded to clinical and genetic information. A third senior adjudicator resolved any significant discrepancies, which were predefined as a difference of >1 point on ordinal scales or disagreement on binary markers.

Individual CSVD lesion definitions adhered to the STRIVE-2 neuroimaging standards (1). Specifically:

■ Lacunar infarcts were defined as round or ovoid lesions of 3–15 mm, with cerebrospinal fluid-like hypointensity on T2-FLAIR and no diffusion restriction.
■ Cerebral microbleeds were defined as focal hypointensities on SWI ≤ 10 mm.
■ Enlarged perivascular spaces (EPVS) were defined as fluid-filled spaces following the course of penetrating vessels.
■ White matter hyperintensities (WMH) were defined as bilateral patchy or confluent hyperintensities on T2-FLAIR.

**Figure 1:**
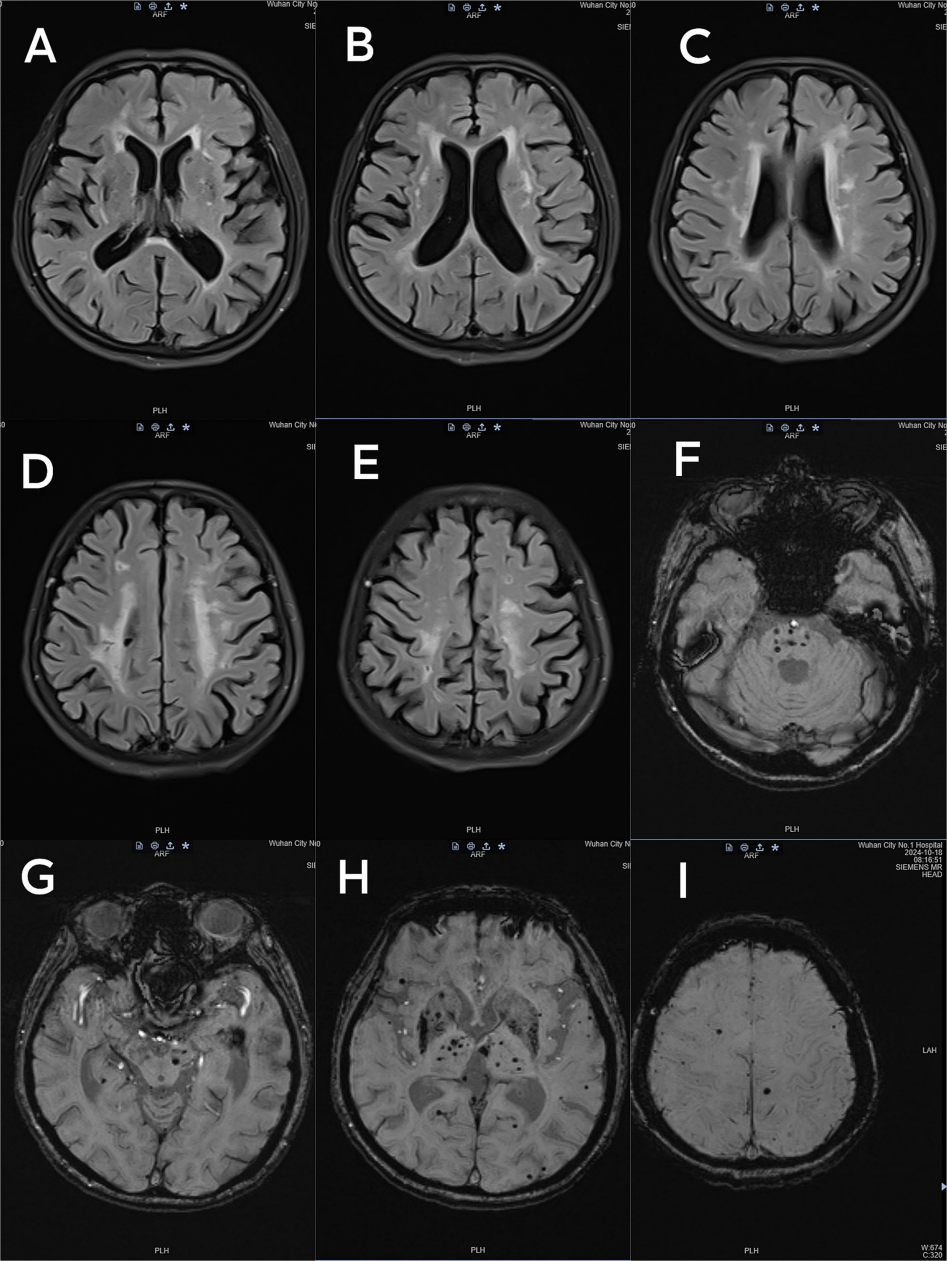
Multi-sequence cranial MRI findings of a 66-year-old female cerebral small vessel disease (CSVD) patient carrying the APOE ε4 allele. **(A–F)** Axial FLAIR images showing multiple lacunar infarcts (some with encephalomalacia) in the bilateral basal ganglia, centrum semiovale, and bilateral frontoparietal-occipital lobes; symmetric confluent T2/FLAIR hyperintensities in periventricular and deep white matter regions (Fazekas score 3); and no enlarged perivascular spaces (EPVS) exceeding 20 in number. **(G–I)** Axial SWI images showing multiple punctate hypointensities (≥5 cerebral microbleeds) in the bilateral basal ganglia, periventricular regions, and frontotemporal-occipital lobe. Scoring according to the study-specific CSVD burden system: Lacunar infarcts (≥1 lesion) → 1 point; White matter hyperintensities (Fazekas score 3) → 2 points; EPVS (number ≤20) → 0 points; Cerebral microbleeds (≥5 lesions) → 2 points. Important clarification: Individual lesion definitions follow the STRIVE-2 neuroimaging standards (1). However, STRIVE-2 does not provide a validated weighted composite burden score. The 0–6 point composite score and its weighting scheme used here are study-specific, adapted from previously published CSVD burden frameworks (10, 11), and not a STRIVE-2-standardized instrument.

STRIVE-2 does not provide a validated weighted composite burden score. Therefore, to quantify the global CSVD burden, we adopted a study-specific composite score (range 0–6), adapted from previously published CSVD burden frameworks (10, 11). This composite score is not a STRIVE-2-standardized instrument. A representative case illustrating the application of this scoring system is shown in Figure 1. It integrated the following chronic markers:

- Lacunar infarction: presence of ≥1 lesion → 1 point
- Cerebral microbleeds: 1–4 lesions → 1 point; ≥5 lesions → 2 points
- Enlarged perivascular spaces (EPVS): number >20 globally → 1 point
- White matter hyperintensities (Fazekas scale): grade 2 → 1 point; grade 3 → 2 points

Global WMH burden was the sum of periventricular and deep WMH Fazekas scores (each 0–3). Periventricular and deep WMH were analyzed separately as secondary outcomes.

Methodological considerations for EPVS assessment.

EPVS were dichotomized globally (>20 vs. ≤20) without regional stratification (e.g., basal ganglia vs. centrum semiovale), as the low prevalence of extensive EPVS precluded reliable regional subgroup analysis. This approach may have reduced sensitivity, because STRIVE-2 emphasizes distinct pathophysiological implications of EPVS topography.

### Inter-rater reliability

Inter-rater reliability was assessed for all markers using the independent ratings of the two neuroradiologists (prior to adjudication). Agreement was quantified using Cohen’s kappa (for EPVS >20 vs. ≤20), quadratic-weighted kappa (for ordinal markers: CMB grades, WMH Fazekas total, periventricular WMH, and deep WMH), and the intraclass correlation coefficient (ICC) with a two-way random model for absolute agreement (single measures) for the global CSVD burden score. As presented in Supplementary Table S1, agreement was excellent for all markers (*κ*/ICC range: 0.899–0.981; all *p* < 0.001), supporting the reproducibility of the neuroimaging assessments.

### Statistical analysis

All statistical analyses were performed using SPSS version 27.0 (IBM Corp., Armonk, NY, United States). A two-tailed *p* value < 0.05 was considered statistically significant.

#### Descriptive statistics

Continuous variables are presented as mean ± standard deviation or median (interquartile range) depending on normality assessed by the Shapiro–Wilk test. Categorical variables are reported as frequencies (percentages). Group comparisons between APOE ε4 carriers and non-carriers were performed using the independent-samples *t*-test or Mann–Whitney U test for continuous variables, and the chi-square test for categorical variables.

#### Main analyses

Binary logistic regression was used to examine the association between APOE ε4 carrier status and each CSVD imaging marker: global CSVD burden score, white matter hyperintensity Fazekas scores (total, periventricular, deep), cerebral microbleed grades, and enlarged perivascular spaces (EPVS, >20 vs. ≤20). For EPVS (prevalence 3.1%, *n* = 22), due to the low event rate, Firth’s penalized logistic regression was applied to reduce sparse-data bias. All models were adjusted for pre-specified covariates: age, sex, hypertension, diabetes mellitus, hyperlipidemia, heart disease, and body mass index. Results are reported as odds ratios (ORs) with 95% confidence intervals (CIs).

#### Effect modification analysis

To investigate whether APOE ε4 modifies the associations between traditional vascular risk factors (hypertension, smoking, alcohol use) and CSVD outcomes, multiplicative interaction terms (e.g., hypertension × APOE ε4) were added to the fully adjusted main effect models. To account for multiple comparisons, *p* values for interactions were adjusted using the Holm-Bonferroni method. Interactions with adjusted *p* < 0.05 were considered statistically significant. For significant interactions, stratified analyses by APOE ε4 carrier status were planned to estimate simple effects. All interaction findings with raw *p* < 0.05 but adjusted *p* > 0.05 are reported as hypothesis-generating.

## Results

### Baseline characteristics of the study population

Baseline characteristics of the study population stratified by APOE ε4 carrier status are summarized in Table 1. Of the 728 included patients, 139 (19.1%) were APOE ε4 carriers (126 ε3/ε4 and 13 ε4/ε4) and 589 (80.9%) were non-carriers. The two groups were well balanced with respect to age (median 70 years in both groups, *p* = 0.996) and sex (48.9% male overall; 49.1% in non-carriers vs. 48.2% in carriers, *p* = 0.854). Body mass index was slightly but significantly lower in APOE ε4 carriers (median 22.72 kg/m^2^) compared with non-carriers (23.51 kg/m^2^, *p* = 0.011). The prevalence of traditional vascular risk factors, including hypertension, diabetes mellitus, hyperlipidemia, current smoking, current alcohol use, and heart disease, did not differ significantly between the two genetic subgroups (all *p* > 0.05). These findings indicate that, apart from a modest difference in BMI, the APOE ε4 carriers and non-carriers were comparable in terms of demographic characteristics and major vascular risk factors, supporting the validity of subsequent multivariable adjustments.

**Table 1:** Baseline characteristics of the study population stratified by APOE ε4 carrier status.

Variable	Total (*N* = 728)	ε4 Non-carriers (*n* = 589)	ε4 Carriers (*n* = 139)	*p-*value
		*n* (ε2/ε2) = 11; *n* (ε2/ε3) = 95; *n* (ε3/ε3) = 483	*n* (ε3/ε4) = 126; *n* (ε4/ε4) = 13	
Demographic characteristics				
Age, years	70(63, 75)	70 (63, 75)	70 (62.76)	0.996
Male, *n* (%)	356 (48.9)	289 (49.1)	67 (48.2)	0.854
BMI(kg/㎡)	23.44 (21.23, 25.39)	23.51 (21.37, 25.53)	22.72 (20.66, 25.07)	**0.011**
Vascular risk factors				
Heart disease, *n* (%)	184 (25.3)	145 (24.6)	39 (28.1)	0.401
Hypertension, *n* (%)	473 (65.0)	389 (66.0)	84 (60.4)	0.212
Diabetes mellitus, *n* (%)	196 (26.9)	160 (27.2)	36 (25.9)	0.762
Hyperlipidemia, *n* (%)	276 (37.9)	226 (38.4)	50 (36.0)	0.600
Current smoking, *n* (%)	229 (31.5)	185 (31.4)	44 (31.7)	0.955
Current alcohol use, *n* (%)	149(20.5)	119 (20.2)	30 (21.6)	0.717

Data are presented as median (interquartile range) for continuous variables, with comparisons made using the Mann–Whitney U test; and as number (percentage) for categorical variables, with comparisons made using the *χ*^2^ test or Fisher’s exact test, as appropriate. All *p*‑values were derived from two‑sided tests. Bold values indicate *p* < 0.05, and *p* < 0.05 was regarded as statistically significant.

### Association of APOE ε4 with CSVD imaging markers

As summarized in Table 2, after full adjustment for demographic characteristics and traditional vascular risk factors, APOE ε4 carrier status was significantly associated with higher periventricular white matter hyperintensity (WMH) burden (*β* = 0.411, 95% CI: 0.046–0.776, *p* = 0.027). No significant associations were observed for the global CSVD burden score (*β* = 0.173, 95% CI: −0.127 to 0.552, *p* = 0.220), global WMH burden (*β* = 0.332, 95% CI: −0.002 to 0.665, *p* = 0.052), deep WMH burden (*β* = 0.236, 95% CI: −0.119 to 0.591, *p* = 0.192), cerebral microbleeds (*β* = −0.050, 95% CI: −0.416 to 0.316, *p* = 0.788), or extensive enlarged perivascular spaces (OR = 1.790, 95% CI: 0.786–3.780, *p* = 0.158). Collectively, these findings suggest that the APOE ε4 allele may have a selective association with periventricular white matter injury, but not with global CSVD burden or other individual imaging markers in this cohort.

**Table 2:** Association of APOE ε4 carrier status with CSVD imaging markers.

Outcome/variable	β / OR	*P* value	95% confidence interval	
			Lower bound	Upper bound
Total CSVD burden	0.173	0.220	−0.127	0.552
Global white matter hyperintensity burden	0.332	0.052	−0.002	0.665
Periventricular white matter hyperintensity burden	0.411	**0.027**	0.046	0.776
Deep white matter hyperintensity burden	0.236	0.192	−0.119	0.591
CMBs	−0.050	0.788	−0.416	0.316
EPVS	1.790	0.158	0.786	3.780

CSVD, cerebral small vessel disease; WMH, white matter hyperintensities; CMBs, cerebral microbleeds; EPVS, enlarged perivascular spaces; OR, odds ratio; CI, confidence interval. ^a^All models were adjusted for age, sex, body mass index, hypertension, diabetes mellitus, hyperlipidemia, heart disease, smoking, and alcohol use. ^b^For total CSVD burden (0–6), global WMH Fazekas total (0–3), periventricular WMH (0–3), deep WMH (0–3), and CMB grades (0/1/2), ordinal logistic regression was used. The reported values are regression coefficients (β). ^c^For EPVS (>20 vs. ≤20), Firth‘s penalized logistic regression was applied due to the low prevalence (4.4%) of the extensive EPVS phenotype. The reported value is odds ratio (OR). ^d^All *p* values are two-sided; *p* < 0.05 was considered statistically significant. All p‑values were derived from two‑sided tests. Bold values indicate *p* < 0.05, and *p* < 0.05 was regarded as statistically.

### Effect modification of traditional vascular risk factors by APOE ε4

Based on exploratory interaction analyses of all candidate risk factors (see Supplementary Table 2), only current smoking and current alcohol use (unadjusted *p* < 0.05) were retained for formal interaction testing; other risk factors were included as covariates only.

Given the limited number of extensive EPVS cases (*n* = 32, 4.4% of the cohort), formal interaction analyses between APOE ε4 and vascular risk factors were not performed. As shown in Supplementary Table 3, EPVS positivity was distributed as follows: 23 cases (3.9%) among non-carriers and 9 cases (6.5%) among ε4 carriers.

We examined multiplicative interactions between APOE ε4 and lifestyle factors (smoking and alcohol use) on CSVD imaging markers. As shown in Table 3, after Holm-Bonferroni correction, the interaction term for ε4 × smoking was statistically significant for global CSVD burden (*β* = 1.977, 95% CI: 1.069–2.884, adjusted *p* < 0.001), CMBs (*β* = 1.926, 95% CI: 0.926–2.926, adjusted *p* < 0.001), total WMH (*β* = 1.280, 95% CI: 0.402–2.157, adjusted *p* = 0.009), and PWMH (*β* = 1.371, 95% CI: 0.421–2.320, adjusted *p* = 0.009), but not for DWMH (adjusted *p* = 0.051). For ε4 × alcohol use, significant interactions were observed for global CSVD burden (*β* = −1.418, 95% CI: −2.439 to −0.397, adjusted *p* = 0.006) and CMBs (*β* = −1.614, 95% CI: −2.733 to −0.494, adjusted *p* = 0.005), but not for WMH or its subtypes (all adjusted *p* > 0.05). These findings indicate that the associations of smoking and alcohol use with specific CSVD phenotypes may be modified by APOE ε4 carrier status.

**Table 3:** Interaction of APOE ε4 with smoking and alcohol use.

CSVD outcome	Interaction term	β/OR	*p* value	95% confidence interval		Adjusted *p* value	Wald *χ*^2^ (df = 1)
				Lower bound	Upper bound		
CMBS	ε4Carrier*Smoking	1.926	0.0002	0.926	2.926	<0.0001	14.25
	ε4Carrier* Alcohol	−1.614	0.0047	−2.733	−0.494	0.005	7.99
DWMH Fazekas	ε4Carrier*Smoking	0.471	0.3173	0.127	1.974	0.051	1
	ε4Carrier* Alcohol	−0.399	0.454	−1.444	0.647	0.909	0.56
PWMH Fazekas	ε4Carrier*Smoking	1.371	0.0046	0.421	2.320	0.009	8.01
	ε4Carrier* Alcohol	−0.460	0.4016	−1.534	0.614	0.401	0.7
WMH Fazekas total	ε4Carrier*Smoking	1.280	0.0043	0.402	2.157	0.009	8.17
	ε4Carrier* Alcohol	−0.469	0.353	−1.458	0.520	0.352	0.86
Global CSVD burden	ε4Carrier*Smoking	1.977	<0.0001	1.069	2.884	<0.0001	18.22
	ε4Carrier* Alcohol	−1.418	0.0065	−2.439	−0.397	0.006	7.4

CSVD, cerebral small vessel disease; CMBs, cerebral microbleeds; DWMH, deep white matter hyperintensities; PWMH, periventricular white matter hyperintensities; WMH, white matter hyperintensities; OR, odds ratio; CI, confidence interval. ^a^All models were adjusted for age, sex, body mass index, hypertension, diabetes mellitus, hyperlipidemia, heart disease, and the other lifestyle factor (smoking or alcohol use). ^b^For global CSVD burden, WMH scores, and CMBs, ordinal logistic regression was used; reported values are regression coefficients (β). ^c^For each CSVD outcome, *p* values for the two interaction terms (ε4 × smoking and ε4 × alcohol use) were adjusted for two comparisons using the Holm-Bonferroni method. Adjusted *p* values are presented in the last column. ^d^An adjusted *p* < 0.05 was considered statistically significant. ^e^*p* values of 0.000 indicate <0.001.

### Sensitivity analyses

To assess the robustness of our findings, we performed a sensitivity analysis by excluding all APOE ε2 carriers and using only ε3/ε3 homozygotes as the reference group.

For the main effects (Supplementary Table 4, Model 1), the association between APOE ε4 and PWMH remained significant (*β* = 0.482, 95% CI: 0.109–0.856, *p* = 0.011), while no significant associations were observed for other CSVD markers (all *p* > 0.05), consistent with the primary results (Table 2).

For the interaction analyses (Supplementary Table 4, Model 2), the interaction effects of APOE ε4 with smoking and alcohol use on CSVD outcomes remained largely consistent with the primary results (Table 3): significant interactions were still observed for ε4 × smoking on global CSVD burden, CMBs, WMH total, and PWMH, and for ε4 × alcohol use on global CSVD burden and CMBs (all adjusted *p* < 0.05).

Collectively, these sensitivity analyses support the robustness of our main findings.

## Discussion

This study systematically investigated the associations between the APOE ε4 allele and neuroimaging markers of cerebral small vessel disease (CSVD), and further evaluated the effect modification of APOE ε4 on the relationships between traditional vascular risk factors (smoking and alcohol consumption) and CSVD. Our main findings are summarized as follows:

(1) The APOE ε4 allele was independently and positively correlated only with periventricular white matter hyperintensities (PWMH), with no significant associations observed with total CSVD burden, overall white matter hyperintensities (WMH), deep white matter hyperintensities (deep WMH), cerebral microbleeds (CMBs), or enlarged perivascular spaces (EPVS);
(2) APOE ε4 significantly modified the effects of smoking and alcohol consumption on specific CSVD phenotypes, and such modifying effects exhibited phenotype specificity.

### Association between APOE ε4 and imaging markers of CSVD: phenotypic specificity

This study found that APOE ε4 was only significantly associated with PWMH (*β* = 0.411, 95% CI: 0.046–0.776, *p* = 0.027), with no significant correlations observed for total WMH, DWMH, CMBs or EPVS. This finding of phenotypic specificity merits further in-depth investigation.

The periventricular white matter region possesses unique anatomical and pathophysiological characteristics. Located at the interface of cerebrospinal fluid and blood exchange, this area is metabolically active and highly susceptible to ischemia, hypoxia, and hypoperfusion. APOE ε4 may exacerbate the formation of PWMH through multiple mechanisms.

First of all, APOE ε4 is strongly associated with blood–brain barrier (BBB) dysfunction. Both animal experiments and human autopsy studies have demonstrated that carriers of APOE ε4 exhibit damage to capillary pericytes and downregulated expression of tight junction proteins, which increases BBB permeability, subsequently facilitating the extravasation of plasma components and interstitial edema in the white matter, thereby exacerbating periventricular white matter hyperintensities (PWMH) (12, 13). Secondly, APOE ε4 impairs the membrane repair and remyelination capacity of oligodendrocytes by regulating cerebral lipid metabolism and cholesterol transport, thereby increasing the susceptibility of periventricular white matter to ischemic injury (14). In addition, APOE ε4 can also promote the release of pro-inflammatory cytokines (TNF-*α*, IL-1β, IL-6) by activating inflammatory pathways such as NF-κB signaling, thereby exacerbating inflammatory damage to the white matter microenvironment (15, 16).

Notably, the inconsistent findings of PWMH with total WMH and DWMH in this study suggest that APOE ε4 may primarily affect periventricular rather than deep white matter regions. This discrepancy may be associated with the watershed blood supply characteristics of the periventricular region. This area lies at the boundary of the terminal blood supply territories of the middle cerebral artery and the anterior/posterior cerebral arteries, rendering it more susceptible to reduced perfusion pressure and microcirculatory dysfunction. The impairment of microvascular function caused by APOE ε4 may exert a more prominent additive effect in this region. Large-sample analyses from the Rhineland study conducted by Lohner et al. also revealed that APOE ε4 exhibits a more consistent association with periventricular WMH rather than deep WMH, which corroborates our findings (17).

However, we found no significant association between APOE ε4 and CMBs as well as EPVS. In terms of CMBs, this may be attributed to the fact that the major driving factors of CMBs are hypertensive vasculopathy and cerebral amyloid angiopathy (CAA). Although APOE ε4 is correlated with CAA, the anatomical distribution of CMBs in this study (without distinguishing lobar versus deep regions) may have masked such an association. The negative findings we observed regarding EPVS may be attributed to the low positive rate of EPVS (4.4%) and the absence of regional stratification (basal ganglia versus centrum semiovale). As emphasized by STRIVE-2, EPVS carries distinct pathological implications across different brain regions: basal ganglia EPVS is predominantly associated with hypertensive arteriosclerosis, while centrum semiovale EPVS tends to reflect CAA-related pathology. The global binary classification approach we adopted (>20 versus ≤20) may have diluted these anatomy-subtype-specific associations.

### Effect modification of APOE ε4 on the association between smoking and CSVD

This study found that APOE ε4 significantly modifies the associations between smoking and multiple CSVD phenotypes (total burden, CMBs, total WMH, PWMH), and the interactions remained significant after Holm-Bonferroni correction. Specifically, the positive correlations between smoking and the above CSVD markers were stronger among APOE ε4 carriers, indicating a synergistic detrimental effect between smoking and APOE ε4.

This finding has clear biological plausibility. Smoking is a well-recognized inducer of oxidative stress and systemic inflammation (18). A variety of toxic substances in tobacco (such as nicotine, tar and carbon monoxide) can induce vascular endothelial cells to produce reactive oxygen species (ROS), resulting in endothelial dysfunction, platelet activation and imbalance of the coagulation-fibrinolysis system (19, 20). Carriers of APOE ε4 inherently exhibit impaired endogenous antioxidant capacity and heightened susceptibility to lipid peroxidation. The APOE ε4 isoform fails to effectively maintain the lipid homeostasis of neuronal membranes, nor can it suppress excessive microglial activation and the release of pro-inflammatory cytokines to the same extent as APOE ε2/ε3 (21–23). Therefore, against the backdrop of smoking-induced oxidative stress and inflammation, the cerebral microvascular system of APOE ε4 carriers may suffer a “two-hit” injury: on the one hand, smoking directly damages the vascular endothelium; on the other hand, APOE ε4 impairs the body’s repair and defense capabilities. This synergistic effect ultimately manifests as more severe white matter microstructural damage and a tendency toward microbleeds.

From the perspective of clinical intervention, this finding provides an important implication: carriers of APOE ε4 may gain greater benefits from smoking cessation interventions. Although this study adopts a cross-sectional design and cannot establish causal relationships, our results suggest that incorporating APOE genotypes into risk stratification for populations at high risk of smoking may help identify individuals who are most in need of intensive smoking cessation interventions.

### Effect modification of APOE ε4 on the association between alcohol consumption and CSVD

Notably, this study found that APOE ε4 significantly modified the associations between alcohol consumption and total CSVD burden as well as CMBs, with negative interaction effect sizes (*β* values of −1.418 and −1.614, respectively). This indicates that the direction of the association between alcohol drinking and CSVD among APOE ε4 carriers is opposite to that among non-carriers, meaning alcohol intake may exert a potential protective modifying effect on the risk of CSVD in APOE ε4 carriers.

Although this finding came as a surprise, it is not unprecedented. Moderate alcohol consumption is thought to exert cardioprotective effects (24), potentially through mechanisms including improved insulin sensitivity, elevated high-density lipoprotein (HDL), anti-platelet aggregation and anti-inflammatory actions (25–27). Existing studies have shown that alcohol can affect microglial activation and the release of inflammatory factors by upregulating nuclear receptors such as PPAR-*γ* (28). Activation of PPAR-γ can promote the polarization of microglia toward an anti-inflammatory phenotype, suggesting its potential role in regulating neuroinflammation (29, 30). Nevertheless, the regulation of PPAR-γ by APOE4 is not unidirectional. Some studies have observed reduced PPAR-γ levels in astrocytes, while others have detected elevated PPAR-γ mRNA or protein levels in animal models under specific pathological conditions such as high-fat diet feeding, indicating that the regulation of PPAR-γ by APOE4 is context-dependent (31, 32). These findings suggest a potential intersection between the alcohol-PPARγ-microglial inflammatory axis and the APOE4-associated neuroinflammatory pathway. However, direct research evidence is currently lacking regarding whether alcohol interacts with APOE4 at the level of this axis, as well as the direction and conditions of such an interaction.

Nevertheless, we remain cautious about these findings. First, alcohol consumption data in this study were binary (current drinkers versus non-drinkers), with no detailed information collected regarding drinking volume, frequency, or beverage type, making it impossible to differentiate the effects of moderate versus excessive alcohol intake. The detrimental impacts of heavy drinking on cerebrovascular and systemic health have been well-established, and our results should not be interpreted as an endorsement of alcohol consumption (33, 34). Second, this interaction was only significant for the total cerebral small vessel disease (CSVD) burden and cerebral microbleeds (CMBs), but not for white matter hyperintensities (WMH) and their subtypes, indicating strong phenotypic specificity with unclear underlying mechanisms. Finally, given that cross-sectional studies cannot eliminate residual confounding and that alcohol drinking behavior is highly correlated with factors such as smoking and socioeconomic status, these results should be regarded as exploratory observations requiring further validation via prospective cohort studies or Mendelian randomization analyses.

### Interaction effect of APOE ε4 on DWMH and EPVS: why negative?

In this study, the interaction effect of APOE ε4 × smoking on DWMH was not statistically significant after adjustment (adjusted *p* = 0.051, only marginally significant), whereas all interaction terms between alcohol consumption and DWMH as well as EPVS were non-significant. Regarding the marginal findings of DWMH, we hypothesize that pathological drivers in deep white matter regions are predominantly hypertensive microangiopathy (35), while APOE ε4 exerts a more prominent modifying effect on the association between periventricular white matter microstructures and Aβ burden, suggesting that this region may be a vulnerable site for APOE ε4-related pathological effects (36). For EPVS, as noted earlier, the extremely low positive rate (4.4%) and lack of regional stratification constituted major limitations, resulting in severely insufficient statistical power for interaction analyses. This limitation should be addressed in future large-sample, multicenter studies.

## Conclusion

In summary, this study found that APOE ε4 was independently and positively correlated with PWMH, and APOE ε4 significantly modified the effects of smoking and alcohol consumption on specific CSVD phenotypes: smoking exerted a synergistic detrimental effect with APOE ε4, whereas alcohol consumption may have a potential protective modifying effect on APOE ε4 carriers. These findings reveal the dual role of APOE ε4 in CSVD, which possesses both an independent genetic effect and an effect-modifying role on environmental exposures, with both functions exhibiting prominent imaging phenotype specificity.

### Future directions

Future research should be further advanced in the following directions: (1) Conduct large-sample prospective cohort studies to verify the longitudinal effects of the interaction between APOE ε4 and smoking/alcohol consumption on the progression of cerebral small vessel disease (CSVD) and cognitive decline; (2) Adopt refined exposure assessments (such as pack-years of smoking, alcohol intake volume and types) to elucidate dose–response relationships; (3) Utilize multimodal imaging (e.g., diffusion tensor imaging (DTI), perfusion imaging) and plasma biomarkers (e.g., p-tau, Aβ, NfL) to explore the molecular pathways underlying the interactions between APOE ε4 and diverse environmental exposures; (4) Perform Mendelian randomization studies on gene–environment interactions to provide genetic evidence for causal relationships.

### Innovation

This study has the following limitations, which should be taken into account when interpreting the results.

First, this study adopts a cross-sectional design, which cannot establish a causal relationship between APOE ε4 and CSVD markers, nor can it infer the temporal sequence of the association between smoking, alcohol consumption and CSVD. All interaction findings should be regarded as correlational rather than causal inferences.

Second, the low positive rate of EPVS (32 cases, 4.4%) substantially limited the statistical power of analyses related to this marker. Accordingly, we did not conduct interaction analyses for EPVS, and the confidence interval for the main effect of EPVS was wide (OR = 1.79, 95% CI: 0.79–3.78), so the results should be interpreted with caution. In addition, EPVS was not stratified and assessed by anatomical regions (basal ganglia versus centrum semiovale), which may obscure CAA-specific associations related to APOE ε4.

Third, only binary information on “current alcohol drinking” was collected for alcohol consumption behavior, with no data obtained on drinking volume, frequency or beverage type. This may lead to imprecise evaluation of alcohol-related effects and residual confounding. Although smoking was recorded as a binary variable, smoking quantity and duration were not differentiated, restricting analyses of dose–response relationships.

Fourth, all participants in this study were CSVD patients with a history of lacunar infarction, so caution is required when generalizing the study conclusions to the general population or other CSVD subtypes. The association patterns between APOE ε4 and CSVD may differ among patients with acute stroke or community-dwelling elderly individuals.

Fifth, although Holm-Bonferroni correction was applied to the *p*-values of interaction terms (2 comparisons per outcome), no multiple comparison correction was performed for main effect analyses. We explicitly defined main effect results with *p* < 0.05 as “nominally significant” and maintained a cautious stance in the discussion section.

## Limitations

This study has the following limitations, which should be taken into account when interpreting the results.

First, this study adopts a cross-sectional design, which cannot establish a causal relationship between APOE ε4 and CSVD markers, nor can it infer the temporal sequence of the association between smoking, alcohol consumption and CSVD. All interaction findings should be regarded as correlational rather than causal inferences.

Second, the low positive rate of EPVS (32 cases, 4.4%) substantially limited the statistical power of analyses related to this marker. Accordingly, we did not conduct interaction analyses for EPVS, and the confidence interval for the main effect of EPVS was wide (OR = 1.79, 95% CI: 0.79–3.78), so the results should be interpreted with caution. In addition, EPVS was not stratified and assessed by anatomical regions (basal ganglia versus centrum semiovale), which may obscure CAA-specific associations related to APOE ε4.

Third, only binary information on “current alcohol drinking” was collected for alcohol consumption behavior, with no data obtained on drinking volume, frequency or beverage type. This may lead to imprecise evaluation of alcohol-related effects and residual confounding. Although smoking was recorded as a binary variable, smoking quantity and duration were not differentiated, restricting analyses of dose–response relationships.

Fourth, all participants in this study were CSVD patients with a history of lacunar infarction, so caution is required when generalizing the study conclusions to the general population or other CSVD subtypes. The association patterns between APOE ε4 and CSVD may differ among patients with acute stroke or community-dwelling elderly individuals.

Fifth, although Holm-Bonferroni correction was applied to the *p*-values of interaction terms (2 comparisons per outcome), no multiple comparison correction was performed for main effect analyses. We explicitly defined main effect results with *p* < 0.05 as “nominally significant” and maintained a cautious stance in the discussion section.

### An integrated model of APOE ε4 in CSVD pathogenesis

Through multi-level systematic analyses, this study ultimately constructs an integrated model elucidating the role of APOE ε4 in cerebral small vessel disease. This model reveals that APOE ε4 functions not as a unidirectional risk factor, but as a dynamic effect modulator. Its impact is dual-faceted: it confers direct genetic susceptibility to specific pathological processes while simultaneously amplifying pathological damage in the presence of specific environmental exposures. This dual-role model not only provides a scientific explanation for the complex phenotypic heterogeneity observed in CSVD imaging but also successfully bridges individual genetic background with conventional vascular risk management within a unified framework.

### Scientific implications and future directions

The scientific implication of this study is that the understanding of CSVD etiology must transition from an isolated “gene *or* environment” perspective to an integrative “gene *and* environment” paradigm. Our findings strongly suggest that future strategies for individualized CSVD risk assessment and prevention should concurrently integrate genetic markers (e.g., APOE ε4) with personalized environmental risk profiles. This paradigm not only provides direct evidence for implementing genetics-based precision primary prevention of CSVD in community populations but also establishes a clear direction for subsequent fundamental research aimed at elucidating the underlying molecular mechanisms. Ultimately, translating this knowledge from the laboratory to clinical practice holds the promise of achieving more effective containment of CSVD as a major threat to brain health.

## Data Availability

The raw data supporting the conclusions of this article will be made available by the authors, without undue reservation.

## References

[1] Duering M Biessels GJ Brodtmann A Chen C Cordonnier C De Leeuw FE . Neuroimaging standards for research into small vessel disease-advances since 2013. Lancet Neurol. (2023) 22:602–18. doi: 10.1016/S1474-4422(23)00131-X, 37236211

[2] Pantoni L. Cerebral small vessel disease: from pathogenesis and clinical characteristics to therapeutic challenges. Lancet Neurol. (2010) 9:689–701. doi: 10.1016/S1474-4422(10)70104-6, 20610345

[3] Brickman AM Siedlecki KL Muraskin J Manly JJ Luchsinger JA Yeung LK . White matter hyperintensities and cognition: testing the reserve hypothesis. Neurobiol Aging. (2011) 32:1588–98. doi: 10.1016/j.neurobiolaging.2009.10.013, 19926168 PMC2891625

[4] Stampfer MJ. Cardiovascular disease and Alzheimer’s disease: common links. J Intern Med. (2006) 260:211–23. doi: 10.1111/j.1365-2796.2006.01687.x, 16918818

[5] Lyall DM Muñoz Maniega S Harris SE Bastin ME Murray C Lutz MW . APOE/TOMM40 genetic loci, white matter hyperintensities, and cerebral microbleeds. Int J Stroke. (2015) 10:1297–300. doi: 10.1111/ijs.12615, 26310205 PMC4950052

[6] Kim J Basak JM Holtzman DM. The role of apolipoprotein E in Alzheimer’s disease. Neuron. (2009) 63:287–303. doi: 10.1016/j.neuron.2009.06.026, 19679070 PMC3044446

[7] Suri S Heise V Trachtenberg AJ Mackay CE. The forgotten APOE allele: a review of the evidence and suggested mechanisms for the protective effect of APOE ɛ2. Neurosci Biobehav Rev. (2013) 37:2878–86. doi: 10.1016/j.neubiorev.2013.10.010, 24183852

[8] Serrano-Pozo A Das S Hyman BT. APOE and Alzheimer’s disease: advances in genetics, pathophysiology, and therapeutic approaches. Lancet Neurol. (2021) 20:68–80. doi: 10.1016/S1474-4422(20)30412-9, 33340485 PMC8096522

[9] Schilling S DeStefano AL Sachdev PS Choi SH Mather KA DeCarli CD . APOE genotype and MRI markers of cerebrovascular disease: systematic review and meta-analysis. Neurology. (2013) 81:292–300. doi: 10.1212/WNL.0b013e31829bfda4, 23858411 PMC3770168

[10] Klarenbeek P van Oostenbrugge RJ Rouhl RPW Knottnerus ILH Staals J. Ambulatory blood pressure in patients with lacunar stroke. Stroke. (2013) 44:2995–9. doi: 10.1161/STROKEAHA.113.002545, 23982717

[11] Staals J Makin SDJ Doubal FN Dennis MS Wardlaw JM. Stroke subtype, vascular risk factors, and total MRI brain small-vessel disease burden. Neurology. (2014) 83:1228–34. doi: 10.1212/WNL.0000000000000837, 25165388 PMC4180484

[12] Nishitsuji K Hosono T Nakamura T Bu G Michikawa M. Apolipoprotein E regulates the integrity of tight junctions in an isoform-dependent manner in an in vitro blood-brain barrier model. J Biol Chem. (2011) 286:17536–42. doi: 10.1074/jbc.M111.225532, 21471207 PMC3093828

[13] Jackson RJ Meltzer JC Nguyen H Commins C Bennett RE Hudry E . APOE4 derived from astrocytes leads to blood-brain barrier impairment. Brain. (2022) 145:3582–93. doi: 10.1093/brain/awab478, 34957486 PMC9586546

[14] Windham IA Powers AE Ragusa JV Wallace ED Zanellati MC Williams VH . APOE traffics to astrocyte lipid droplets and modulates triglyceride saturation and droplet size. J Cell Biol. (2024) 223:e202305003. doi: 10.1083/jcb.202305003, 38334983 PMC10857907

[15] Friedberg JS Aytan N Cherry JD Xia W Standring OJ Alvarez VE . Associations between brain inflammatory profiles and human neuropathology are altered based on apolipoprotein E ε4 genotype. Sci Rep. (2020) 10:2924. doi: 10.1038/s41598-020-59869-5, 32076055 PMC7031423

[16] Rawat V Wang S Sima J Bar R Liraz O Gundimeda U . ApoE4 alters ABCA1 membrane trafficking in astrocytes. J Neurosci. (2019) 39:9611–22. doi: 10.1523/JNEUROSCI.1400-19.2019, 31641056 PMC6880458

[17] Lohner V Pehlivan G Sanroma G Miloschewski A Schirmer MD Stöcker T . Relation between sex, menopause, and White matter Hyperintensities: the Rhineland study. Neurology. (2022) 99:e935–43. doi: 10.1212/WNL.0000000000200782, 35768207 PMC9502737

[18] Durazzo TC Mattsson N Weiner MW. Smoking and increased Alzheimer’s disease risk: a review of potential mechanisms. Alzheimers Dement. (2014) 10:S122–45. doi: 10.1016/j.jalz.2014.04.009 24924665 PMC4098701

[19] Münzel T Crea F Rajagopalan S Lüscher T. Nicotine and the cardiovascular system: unmasking a global public health threat. Eur Heart J. (2025) 47:1764–81. doi: 10.1093/eurheartj/ehaf1010, 41406987 PMC13079999

[20] Mallah MA Soomro T Ali M Noreen S Khatoon N Kafle A . Cigarette smoking and air pollution exposure and their effects on cardiovascular diseases. Front Public Health. (2023) 11:967047. doi: 10.3389/fpubh.2023.967047, 38045957 PMC10691265

[21] Iannucci J Sen A Grammas P. Isoform-specific effects of apolipoprotein E on markers of inflammation and toxicity in brain glia and neuronal cells in vitro. Curr Issues Mol Biol. (2021) 43:215–25. doi: 10.3390/cimb43010018, 34071762 PMC8928993

[22] Lynch JR Tang W Wang H Vitek MP Bennett ER Sullivan PM . APOE genotype and an ApoE-mimetic peptide modify the systemic and central nervous system inflammatory response. J Biol Chem. (2003) 278:48529–33. doi: 10.1074/jbc.M306923200, 14507923

[23] Ralhan I Do AD Bae JY Feringa FM Cai W Chang J . Protective ApoE variants support neuronal function by effluxing oxidized phospholipids. Neuron. (2026) 114:661–678.e10. doi: 10.1016/j.neuron.2025.10.040, 41338186

[24] Xiong BB Wang ZJ Li ZM Yang TN Li XY Lu MJ . Association between alcoholic beverage consumption and cerebral small vessel disease burden. J Prev Alzheimers Dis. (2025) 12:100322. doi: 10.1016/j.tjpad.2025.100322, 40754514 PMC12627891

[25] O’Keefe JH Bybee KA Lavie CJ. Alcohol and cardiovascular health: the razor-sharp double-edged sword. J Am Coll Cardiol. (2007) 50:1009–14. doi: 10.1016/j.jacc.2007.04.089, 17825708

[26] Martínez-González MÁ Hernández Hernández A. Effect of the Mediterranean diet in cardiovascular prevention. Rev Esp Cardiol (Engl Ed). (2024) 77:574–82. doi: 10.1016/j.rec.2024.01.006, 38336153

[27] Li X Hur J Cao Y Song M Smith-Warner SA Liang L . Moderate alcohol consumption, types of beverages and drinking pattern with cardiometabolic biomarkers in three cohorts of US men and women. Eur J Epidemiol. (2023) 38:1185–96. doi: 10.1007/s10654-023-01053-w, 37747628 PMC10924636

[28] Li C Li J Loreno EG Miriyala S Panchatcharam M Lu X . Chronic low-dose alcohol consumption attenuates post-ischemic inflammation via PPARγ in mice. Int J Mol Sci. (2021) 22:5121. doi: 10.3390/ijms22105121, 34066125 PMC8150922

[29] Guo S Wang H Yin Y. Microglia polarization from M1 to M2 in neurodegenerative diseases. Front Aging Neurosci. (2022) 14:815347. doi: 10.3389/fnagi.2022.815347, 35250543 PMC8888930

[30] García-Baos A Alegre-Zurano L Cantacorps L Martín-Sánchez A Valverde O. Role of cannabinoids in alcohol-induced neuroinflammation. Prog Neuro-Psychopharmacol Biol Psychiatry. (2021) 104:110054. doi: 10.1016/j.pnpbp.2020.110054, 32758518

[31] Blumenfeld J Yip O Kim MJ Huang Y. Cell type-specific roles of APOE4 in Alzheimer disease. Nat Rev Neurosci. (2024) 25:91–110. doi: 10.1038/s41583-023-00776-9, 38191720 PMC11073858

[32] Rai A Ojiakor OA Rylett RJ. Detection of early Alzheimer’s disease-like molecular alterations in a mouse model expressing human ApoE4. J Neurochem. (2023) 166:572–87. doi: 10.1111/jnc.15904, 37415276

[33] HindsholmMF DasAS GokcalE MorottiA RotschildO SimonsenCZ . Effects of heavy alcohol use on acute intracerebral hemorrhage and cerebral small vessel disease. Neurology. (2025) 105:e214348. doi: 10.1212/WNL.0000000000214348, 41191856

[34] Hisamatsu T Tabara Y Kadota A Torii S Kondo K Yano Y . Alcohol consumption and cerebral small- and large-vessel diseases: a Mendelian randomization analysis. J Atheroscler Thromb. (2024) 31:135–47. doi: 10.5551/jat.64222, 37612092 PMC10857837

[35] Santisteban MM Iadecola C Carnevale D. Hypertension, neurovascular dysfunction, and cognitive impairment. Hypertension. (2023) 80:22–34. doi: 10.1161/HYPERTENSIONAHA.122.18085, 36129176 PMC9742151

[36] Chen CL Son SJ Schweitzer N Jin H Li J Wang L . Periventricular diffusivity reflects APOE ε4-modulated amyloid accumulation and cognitive impairment in the Alzheimer’s disease continuum. Alzheimers Dement. (2025) 21:e70659. doi: 10.1002/alz.70659, 40965291 PMC12444947

